# Associations between occupational and environmental exposures and organ involvement in sarcoidosis: a retrospective case-case analysis

**DOI:** 10.1186/s12931-021-01818-5

**Published:** 2021-08-09

**Authors:** Steven Ronsmans, Jolien De Ridder, Eline Vandebroek, Stephan Keirsbilck, Benoit Nemery, Peter H. M. Hoet, Steven Vanderschueren, Wim A. Wuyts, Jonas Yserbyt

**Affiliations:** 1 Clinic for Occupational and Environmental Medicine, Department of Respiratory Diseases, University Hospitals Leuven, Leuven, Belgium; 2 Centre for Environment and Health, Department of Public Health and Primary Care, KU Leuven, Leuven, Belgium; 3 Unit for Interstitial Lung Diseases, Department of Respiratory Diseases, University Hospitals Leuven, Herestraat 49, 3000 Leuven, Belgium; 4 Premed, External Service for Prevention and Protection at Work, Leuven, Belgium; 5 IDEWE, External Service for Prevention and Protection at Work, Leuven, Belgium; 6 Department of General Internal Medicine, University Hospitals Leuven, Leuven, Belgium; 7 Laboratory of Clinical Infectious and Inflammatory Diseases, Department of Microbiology, Immunology, and Transplantation, KU Leuven, Leuven, Belgium; 8 Laboratory of Respiratory Diseases and Thoracic Surgery (BREATHE), Department CHROMETA, KU Leuven, Leuven, Belgium

**Keywords:** Sarcoidosis phenotypes, Extrapulmonary sarcoidosis, Occupational and environmental exposures

## Abstract

**Background:**

Sarcoidosis most commonly affects lungs and intrathoracic lymph nodes, but any other organ can be involved. In epidemiological studies, many occupational and environmental exposures have been linked to sarcoidosis but their relationship with the disease phenotype has barely been studied.

**Objective:**

To investigate how occupational and environmental exposures prior to diagnosis relate to organ involvement in patients with sarcoidosis

**Methods:**

We retrospectively studied patients seen at a sarcoidosis clinic between 2017 and 2020. Patients were included if they had a clinical presentation consistent with sarcoidosis and histologically confirmed epithelioid granulomas or had Löfgren syndrome. In a case–case analysis using multivariable logistic regression we calculated odds ratios (OR) of prespecified exposure categories (based on expert ascertainment) for cases with a given organ involvement versus cases without this organ involvement.

**Results:**

We included 238 sarcoidosis patients. Sarcoidosis limited to pulmonary involvement was associated with exposure to inorganic dust prior to diagnosis (OR 2.11; 95% confidence interval [CI] 1.11–4.17). Patients with liver involvement had higher odds of contact with livestock (OR 3.68; 95% CI 0.91–12.7) or having jobs with close human contact (OR 4.33; 95% CI 1.57–11.3) than patients without liver involvement. Similar associations were found for splenic involvement (livestock: OR 4.94, 95% CI 1.46–16.1; close human contact: OR 3.78; 95% CI 1.47–9.46). Cardiac sarcoidosis was associated with exposure to reactive chemicals (OR 5.08; 95% CI 1.28–19.2) or livestock (OR 9.86; 95% CI 1.95–49.0). Active smokers had more ocular sarcoidosis (OR 3.26; 95% CI 1.33–7.79).

**Conclusions:**

Our study indicates that, in sarcoidosis patients, different exposures might be related to different organ involvements—hereby providing support for the hypothesis that sarcoidosis has more than one cause, each of which may promote a different disease phenotype.

**Supplementary Information:**

The online version contains supplementary material available at 10.1186/s12931-021-01818-5.

## Introduction

Sarcoidosis most commonly affects the lungs and intrathoracic lymph nodes, but any organ can be involved [1, 2]. Several lines of evidence support the idea that sarcoidosis results from exposure of susceptible individuals to one or several antigen(s), leading to the activation of macrophages and T-cell immunity against these antigens. In epidemiological studies, many occupational and environmental exposures have been linked to sarcoidosis, such as organic dust [3–5], inorganic dust (including metals and minerals) [6, 7], and infectious agents (including mycobacteria and *Cutibacterium acnes*) [8]*.* It is unclear whether these exposures are truly ‘causing’ the disease, whether they render the immune system more susceptible to the development of sarcoidosis, or whether they exacerbate subclinical cases.

The diverse clinical manifestations and the wide range of associated exposures fuel the hypothesis that sarcoidosis has more than one cause, each of which may promote a different disease phenotype [9]. However, the relationship between exposure and disease phenotype has barely been studied. Indirect support for this hypothesis comes from studies demonstrating distinct patterns of organ involvement in men and women—who have different occupational exposures [10–12]. Also, studies have shown that sarcoidosis patients with respiratory exposure to inorganic or organic dust are less likely to have extrapulmonary involvement than unexposed patients [6, 12, 13].

In this study, we selected a range of occupational/environmental exposures previously associated with sarcoidosis [3–8] and assessed the relationship between these exposures (prior to diagnosis) and organ involvements in patients visiting a sarcoidosis clinic.

## Materials and methods

We retrospectively studied sarcoidosis patients that had visited the outpatient sarcoidosis clinic at the Department of Respiratory Diseases in the University Hospitals Leuven (Belgium) between January 1, 2017 and November 1, 2020 (n = 321) [14]. The clinic is a WASOG Sarcoidosis Centre of Excellence which means that the assessment of patients with sarcoidosis can involve the expertise of pulmonologists, cardiologists, dermatologists, ophthalmologists, rheumatologists and neurologists. All patients were subjected to history taking, clinical examination, laboratory work-up including urinalysis, chest imaging, eye examination, electrocardiography, and lung function testing. Pathological confirmation was sought except in patients presenting with the Löfgren syndrome. Further testing was based on clinical symptoms, signs or abnormalities detected by baseline screening tests in accordance with current guidelines [15].

Patients were included in our study if they had a clinical presentation consistent with sarcoidosis and histologically confirmed epithelioid granulomas (with negative cultures and stains for acid fast bacilli) or had Löfgren syndrome (n = 304) [15]. Patients under the age of 18 (n = 3), with a previous history of malignancy (n = 44), and with missing data on job history (n = 19) were excluded. The study was approved by the Ethics Committee Research UZ/KU Leuven (S64710).

### Outcomes

An organ was considered affected when involvement was “highly probable” or “probable” according to the WASOG Sarcoidosis Organ Assessment Instrument [16]. Skin involvement was divided into “specific” lesions—resulting from the presence of granulomas in the skin—and erythema nodosum. Pulmonary-only sarcoidosis was defined as lung or intrathoracic lymph node involvement, without any evidence of other internal organ involvement (liver, spleen, heart, bone marrow, parotid/salivary gland or neurological system).

### Exposures

Exposure categories were selected based on previously demonstrated associations with sarcoidosis [3–8]. Two experts in occupational and environmental medicine independently estimated how likely patients had been exposed prior to diagnosis (unlikely, probable or possible) based on information on jobs, hobbies and housing conditions extracted from the medical records. The following exposures were assessed: (1) respiratory exposure to reactive chemicals (such as isocyanates, methacrylates, epoxy resins), (2) inorganic dust (including metals and silica) or (3) organic dust (plant, animal, or microbial antigens), if they had (4) close contact with livestock (such as cows, sheep, goats or horses), (5) jobs with close human contact (such as health care professionals, educators, and child or elderly care workers), or (6) administrative jobs. A patient was considered exposed when both experts estimated that there had been at least ‘possible’ exposure, and at least one expert estimated that the exposure was ‘probable’. Multiple exposures could be assigned to one patient. The experts were blinded for any demographic or clinical information.

### Covariates

Covariates which could potentially influence the association between exposures and outcomes were extracted from the medical records: sex, race, age at diagnosis, presence of autoimmune or autoinflammatory diseases (such as Sjögren syndrome, rheumatoid arthritis, psoriasis, inflammatory bowel disease, diabetes mellitus type 1, alopecia areata, vitiligo, etc.), genetic disorders (such as autosomal dominant polycystic kidney disease), a family history of sarcoidosis, a family history of autoimmune or autoinflammatory disease, having metal prostheses or silicone implants, or taking medication (before diagnosis) that could have potentially triggered sarcoidosis (e.g., anti-TNF).

### Statistical analysis

We did a case–case analysis to examine the association between the studied exposures and organ involvements. A case-case analysis is a special form of a case–control analysis in which cases with different subtypes of the same illness are compared instead of including disease-free controls. This approach can reduce selection and recall bias relative to other case–control formats by ensuring that both case and “control” subjects have all been affected by (a different phenotype of) the same disease and thus underwent a similar selection process. First, univariable logistic regression was performed between each organ involvement and each exposure. Subsequently, for each organ involvement a separate multivariable logistic regression model was constructed to investigate statistical associations with the various exposures. The selection of exposures included in the final model for each organ involvement was done using an automated model selection procedure, implemented by the R package *glmulti* [17]*.* The best-fit models were selected based on their AIC ranking (Akaike Information Criterion) among all possible models—considering all possible subsets of exposure variables and other covariates (sex, age, presence of a systemic or organ-specific autoimmune disease, presence of genetic disorders, family history of sarcoidosis, family history of autoimmune or autoinflammatory diseases, having metal prostheses or silicone implants and taking medication that could have potentially triggered sarcoidosis). Additionally, tests for interaction were performed using interaction terms in the logistic regression models. Results were expressed as odds ratios (ORs) with 95% confidence intervals (CI). The OR from this analysis represent the odds of having been exposed for cases with a given organ involvement divided by the odds for all other cases without this organ involvement [18].

### Sensitivity analysis

The case-case analysis does remain vulnerable to selection bias. Because smoking and respiratory exposures to reactive chemicals, inorganic or organic dust might lead to respiratory health effects independently of the presence of sarcoidosis, exposed patients could potentially seek medical care earlier than unexposed. Respiratory symptoms might therefore be potentially confounding the association between exposure and organ involvement. To assess this potential confounding, we performed a sensitivity analysis by adjusting for the different lung function parameters (at diagnosis) in the regression models.

All statistical analyses were performed in statistical computing language R [19]. The STROBE (Strengthening the Reporting of Observational Studies in Epidemiology) guideline was followed for reporting the study [20].

## Results

We included 238 sarcoidosis patients—84 women and 154 men—who were predominantly Caucasian (4 were African, 1 Asian). Median age was 45 years (interquartile range 37–52 year) for women and 41 years (IQR 35–47 year) for men (Table 1). Sarcoidosis limited to lung and/or lymph nodes was present in 164 patients (69%). The most common extrapulmonary organ involvements were spleen (16%), eye (12%), liver (9.7%), and heart (7.1%) (Table 2).Chest CT findings and lung function parameters of the included patients are shown in Table 1. Comorbidities and other covariates are presented in the Additional file 1: Table S1.

**Table 1:** Demographic and clinical data of the included sarcoidosis patients according to exposure category

	Overall (n = 238)	Exposure						
		Reactive chemical (n = 26)	Inorganic dust (n = 74)	Organic dust (n = 63)	Contact with livestock (n = 15)	Close human contact (n = 31)	Admin work only (n = 42)	Active smoker (n = 43)
**Demographics**								
Gender								
Women	84 (35%)	5 (19%)^§^	3 (4.1%)*	22 (35%)	6 (40%)	25 (81%)*	22 (52%)*	9 (21%)*
Men	154 (65%)	21 (81%)^§^	71 (96%)*	41 (65%)	9 (60%)	6 (19%)*	20 (48%)*	34 (79%)*
Age at diagnosis	42 (35–50)	37 (34–49)	41 (35–48)	39 (34–48)^§^	47 (38–52)	44 (39–50)	42 (36–46)	38 (30–46)*
**Smoking**								
Never smoker	148 (62%)	14 (54%)	40 (54%)^§^	43 (68%)	14 (93%)*	21 (68%)	30 (71%)	0 (0%)
Past smoker	47 (20%)	6 (23%)	17 (23%)	13 (21%)	1 (6.7%)	5 (16%)	6 (14%)	0 (0%)
Active smoker	43 (18%)	6 (23%)	17 (23%)	7 (11%)^§^	0 (0%)^§^	5 (16%)	6 (14%)	43 (100%)
Packyears (for past or active smokers)	10 (5–20)	7.5 (5–20)	10 (5–20)	12 (5–20)	45	5 (5–14)	10 (9–15)	10 (6–20)
**Chest CT at diagnosis**								
Enlarged hilar/mediastinal lymph nodes	235 (99%)	25 (96%)	73 (99%)	63 (100%)	15 (100%)	31 (100%)	40 (95%)^§^	43 (100%)
(Micro)nodules	183 (77%)	21 (81%)	55 (74%)	51 (81%)	10 (67%)	22 (71%)	35 (83%)	36 (84%)
Fibrotic changes	13 (5.5%)	2 (7.7%)	4 (5.4%)	2 (3.2%)	0 (0%)	2 (6.5%)	5 (12%)^§^	4 (9.3%)
Airway abnormalities	44 (18%)	7 (27%)	20 (27%)*	9 (14%)	1 (6.7%)	3 (9.7%)	8 (19%)	10 (23%)
**Lung function at diagnosis**								
FVC %pred	94 (85–105)	91 (77–102)	94 (81–103)	90 (78–98)*	94 (91–103)	99 (86–108)	94 (90–108)^§^	94 (81–106)
FEV_1_%pred	89 (76–102)	84 (70–98)	89 (75–104)	82 (71–92)*	92 (87–98)	90 (77–102)	94 (80–106)	81 (70–99)^§^
FEV_1_/FVC%	78 (73–83)	76 (62–82)^§^	77 (74–82)	76 (70–80)*	80 (77–82)	81 (71–85)	80 (76–83)^§^	78 (67–83)
TLC %pred	92 (83–100)	88 (80–99)	89 (80–98)^§^	86 (80–98)^§^	91 (86–97)	94 (89–102)	96 (88–100)^§^	86 (80–101)
T_LCO_ %pred	80 (68–92)	74 (64–93)	81 (67–95)	80 (68–88)	84 (74–96)	81 (68–87)	79 (69–90)	73 (62–89)*
**Broncho-alveolar lavage**								
% Lymphocytes	17 (10–25)	19 (14–25)	15 (10–21)	15 (10–23)	13 (7–33)	18 (11–32)	15 (7–25)	14 (7–21)
Not available	125 (53%)	13 (50%)	34 (46%)	27 (43%)	9 (60%)	20 (65%)	24 (57%)	21 (49%)
**Treatment**								
Treated within first year after diagnosis	118 (50%)	10 (38%)	41 (55%)	33 (52%)	5 (33%)	16 (52%)	21 (50%)	26 (60%)
Oral corticosteroids	116 (49%)	10 (38%)	41 (55%)	33 (52%)	5 (33%)	16 (52%)	19 (45%)	26 (60%)^§^
Methotrexate	46 (19%)	5 (19%)	17 (23%)	14 (22%)	0 (0%)^§^	5 (16%)	7 (17%)	16 (37%)*
Azathioprine	47 (20%)	6 (23%)	17 (23%)	15 (24%)	1 (6.7%)	5 (16%)	10 (24%)	14 (33%)*
Chloroquine	27 (11%)	4 (15%)	12 (16%)	8 (13%)	0 (0%)	3 (9.7%)	4 (9.5%)	10 (23%)*

Statistics presented: median (25–75%), n (%); Statistical tests performed, comparing exposed to non-exposed: Wilcoxon rank sum test, Fisher’s exact test or Pearson’s Chi-squared test; * when p < 0.05, ^§^p < 0.10

**Table 2:** Organ involvements of the included sarcoidosis patients according to exposure category

	Overall (n = 238)	Exposure						
		Reactive chemicals (n = 26)	Inorganic dust (n = 74)	Organic dust (n = 63)	Contact with livestock (n = 15)	Close human contact (n = 31)	Admin work only (n = 42)	Active smoker (n = 43)
**Organ involvement**								
Pulmonary involvement only	164 (69%)	17 (65%)	58 (78%)*	47 (75%)	6 (40%)*	16 (52%)*	27 (64%)	32 (74%)
Intrathoracic lymph node	235 (99%)	25 (96%)	73 (99%)	63 (100%)	15 (100%)	31 (100%)	40 (95%)^§^	43 (100%)
Lung	191 (80%)	22 (85%)	59 (80%)	53 (84%)	11 (73%)	24 (77%)	34 (81%)	38 (88%)
Liver	23 (9.7%)	1 (3.8%)	3 (4.1%)*	8 (13%)	4 (27%)*	8 (26%)*	4 (9.5%)	3 (7.0%)
Spleen	37 (16%)	3 (12%)	6 (8.1%)*	7 (11%)	6 (40%)*	10 (32%)*	9 (21%)	4 (9.3%)
Cardiac	17 (7.1%)	5 (19%)*	3 (4.1%)	2 (3.2%)	4 (27%)*	2 (6.5%)	4 (9.5%)	3 (7.0%)
Eye	29 (12%)	2 (7.7%)	7 (9.5%)	4 (6.3%)^§^	0 (0%)	6 (19%)	5 (12%)	11 (26%)*
Skin (excluding erythema nodosum)	23 (9.7%)	2 (7.7%)	6 (8.1%)	8 (13%)	1 (6.7%)	2 (6.5%)	3 (7.1%)	7 (16%)
Erythema nodosum	30 (13%)	5 (19%)	11 (15%)	9 (14%)	0 (0%)	3 (9.7%)	5 (12%)	4 (9.3%)
Neurologic	9 (3.8%)	1 (3.8%)	2 (2.7%)	1 (1.6%)	0 (0%)	2 (6.5%)	1 (2.4%)	2 (4.7%)
Parotid/salivary gland	8 (3.4%)	0 (0%)	1 (1.4%)	3 (4.8%)	2 (13%)^§^	2 (6.5%)	2 (4.8%)	0 (0%)
Bone marrow	9 (3.8%)	0 (0%)	2 (2.7%)	3 (4.8%)	1 (6.7%)	3 (9.7%)^§^	1 (2.4%)	3 (7.0%)
Löfgren syndrome	30 (13%)	3 (12%)	9 (12%)	6 (10%)	1 (7%)	3 (10%)	6 (14%)	3 (7%)

Statistics presented: n (%); Statistical tests performed, comparing exposed to non-exposed: Fisher’s exact test or Pearson’s Chi-squared test; * when p < 0.05, ^§^p < 0.10

The majority of the patients had never smoked (62%), and only 18% was an active smoker. Twenty-six patients (11%) had been exposed to reactive chemicals (such as isocyanates, methacrylates, epoxy resins) prior to diagnosis, mostly while having jobs in building industry (working with reactive adhesives or epoxy resins) or in chemical industry (for example in a paint factory, plastics/polymers production, or a chemical laboratory). Seventy-four patients (31%) had been exposed to inorganic dust, which included patients with jobs in which they were exposed to metal dust and/or fumes (such as metal workers and welders) or jobs with silica exposure (such as road and building construction workers or plumbers). Sixty-three patients (26%) had had organic dust exposure (plant, animal, or microbial antigens), which included mostly patients working in food production (bakers, cooks, butchers), wood workers, gardeners, farmers and pigeon breeders. Fifteen patients (6.3%) had close contact with mammalian livestock (cows, sheep, goats, or horses), mainly as animal farmers.

Thirty-one patients (13%) had jobs with close human contact, such as health care professionals, educators, and child or elderly care workers. Forty-four patients (18%) had only had administrative jobs. Men were more likely to have had inorganic dust exposure (46% of men versus 3.6% of women), while women were more likely to have jobs with close human contact or administrative jobs (Table 1).

### Associations between organ involvements and exposures

Table 2 shows the distribution of organ involvements for patients in each exposure category. Results of the univariable logistic regression analysis (without adjustment for other exposures or covariates) of the associations between organ involvements and exposures are shown in the Additional file 1: Table S2.

For each organ involvement a multivariable logistic regression model was constructed to investigate statistical associations with the various exposures (Table 3). The selection of exposures included in the final model for each organ involvement was done using an automated model selection procedure, based on AIC ranking, among all possible models—considering all possible subsets of exposure variables and other covariates such as sex and age (see “Materials and methods”). In the final multivariable models, isolated pulmonary sarcoidosis was associated with inorganic dust exposure (OR 2.11; 95% confidence interval [CI] 1.11–4.17) and tended to be associated with organic dust exposure (OR 1.86; 95% CI 0.95–3.82). Sarcoidosis patients with liver involvement had higher odds of having contact with livestock (OR 3.68; 95% CI 0.91–12.7) and having jobs with close human contact (OR 4.33; 95% CI 1.57–11.3) than patients without liver involvement. Splenic involvement was associated with contact with livestock (OR 4.94; 95% CI 1.46–16.1), jobs with close human contact (OR 3.78; 95% CI 1.47–9.46), and with administrative jobs (OR 2.52; 95% CI 0.99–6.16). Cardiac sarcoidosis was associated with exposure to reactive chemicals (OR 5.08; 95% CI 1.28–19.2) and contact with livestock (OR 9.86; 95% CI 1.95–49.0).

**Table 3:** Final multivariable logistic regression models for six organ involvements showing associations with occupational and environmental exposures in patients with sarcoidosis

Exposure	Pulmonary only (n = 164)			Liver involvement (n = 23)			Splenic involvement (n = 37)			Cardiac involvement (n = 17)			Eye involvement (n = 29)			Skin granulomas (n = 23)		
	**OR**	95% CI	p	OR	95% CI	p	OR	95% CI	p	OR	95% CI	p	OR	95% CI	p	OR	95% CI	p
Reactive chemicals (n = 26)		–			–			–		5.08*	1.28–19.2	0.016		–			–	
Inorganic dust (n = 74)	2.11*	1.11–4.17	0.026		–			–		0.21*	0.04–0.76	0.029		–			–	
Organic dust (n = 63)	1.86	0.95–3.82	0.079		–			–		0.22	0.03–0.91	0.066		–			–	
Contact with livestock (n = 15)	0.23*	0.07–0.71	0.012	3.68*	0.91–12.7	0.047	4.94*	1.46–16.1	0.008	9.86*	1.95–49.0	0.004	0.00		> 0.99		–	
Close human contact (n = 31)		–		4.33*	1.57–11.3	0.003	3.78*	1.47–9.46	0.005		–			–			–	
Administrative work only (n = 42)		–			–		2.52*	0.99–6.16	0.046		–			–			–	
Active smoker (n = 43)		–			–			–			–		3.26*	1.33–7.79	0.008	2.50	0.89–6.54	0.069

For each organ involvement a separate multivariable logistic regression model was constructed. Odd ratios shown result from the best-fit multivariable logistic regression models, using Akaike information criterion (AIC) for model selection. OR = Odds Ratio for having been exposed (cases with a given organ involvement versus all other cases) while adjusting for other exposures and covariates retained in the final models (See “Materials and methods”). CI = Confidence Interval; p = p-value; *when statistically significant (p < 0.05)

Active smokers had more ocular sarcoidosis (OR 3.26; 95% CI 1.33–7.79) and were possibly more likely to have skin granulomas (OR 2.50; 95% CI 0.89–6.54). No statistically significant associations were found between erythema nodosum and any exposure (model not shown). A sensitivity analysis including adjustment for lung function at diagnosis yielded similar effect size estimates (see Additional file 1: Table S3).

## Discussion

Our study demonstrated that various occupational/environmental exposures prior to diagnosis affect organ involvement in sarcoidosis patients. The main novelty of our study is that unlike previous studies searching for associations between exposure and sarcoidosis occurrence, we investigated the associations between exposure and *organ involvement* in sarcoidosis—hereby aiming to provide support for the hypothesis that each “cause” of sarcoidosis might promote a different disease phenotype [9].

We selected a range of exposures which had been previously associated with sarcoidosis [3–8]. Due to the wide range of exposures described in the literature, we categorized them in 5 categories: inorganic dust, organic dust, reactive chemicals, contact with (mammalian) livestock, and close human contact. We speculated that the last two categories entailed an increased risk of exposure to infectious agents. A sixth category—“administrative work only”—was used for patients without any of the above-mentioned exposures.

### Dust exposure

We found a significant association between exposure to inorganic dust, such as metal or silica dust, and sarcoidosis limited to lungs and/or intrathoracic lymph nodes (OR 2.11; 95% CI 1.11–4.17). Also, organic dust exposure—including exposure to plant, animal, or microbial antigens—tended to be related to pulmonary-only sarcoidosis (OR 1.86; 95% CI 0.95–3.82).

In the ACCESS (A Case Control Etiologic Study of Sarcoidosis) study, patients exposed to agricultural organic dust (in whites, OR 0.33; 95% CI 0.16–0.71) and wood burning (in blacks, OR 0.36; 95% CI 0.23–0.59) were less likely to have extrapulmonary involvement [12]. World Trade Center (WTC) rescue workers with sarcoidosis—who had been exposed to high levels of inorganic dust resulting from the WTC collapse in 2001—also had less extrapulmonary involvement than expected [6]. Patients with chronic beryllium disease, a disorder clinically, radiologically and histopathologically almost indistinguishable from sarcoidosis—but known to be caused by a cell-mediated immune response to beryllium—have fewer extrapulmonary manifestations: hepatic, splenic and cardiac involvement are rare, and ocular and neurological impairment have not been reported [21].

In sarcoidosis, epithelioid granulomas are presumably an immunological response to persistent antigens, possibly combined with an adjuvant signal triggering an innate immune response [9]. Dust particles might be the target of this immune response, although it is uncertain if they act as antigens, as adjuvants or as nidus [9].

A possible explanation for the association between dust exposure and sarcoidosis which is limited to the lungs is that inhaled dust particles do not readily disseminate systemically. Small inhaled particles that deposit in the deep lung can—when not removed by the mucociliary escalator—be transported via the lymphatic system to regional lymph nodes [22, 23]. Particles accumulate in lymph nodes but can—to a limited extent—gradually translocate into the systemic circulation where they are filtered from the blood in liver and spleen [22]. Small fractions can be taken up by other organs such as the brain or the heart [24]. The probability and speed of systemic dissemination depends on particle characteristics such as size, surface properties, chemical composition and solubility [22]. This might explain why extrapulmonary involvement in patients exposed to dust is less common, but not impossible.

### Reactive chemicals

We found that respiratory exposure to reactive chemicals—including isocyanates, methacrylates, or epoxy resins—was associated with the presence of cardiac sarcoidosis (OR 5.08; 95% CI 1.28–19.2). We should be cautious in interpreting this result as it concerns a limited number of patients (exposure to reactive chemicals was present in 5 out of 17 patients [29%] with cardiac sarcoidosis, but only in 21/221 cases [9.5%] without cardiac involvement). As these chemicals are known to cause asthma (and occasionally hypersensitivity pneumonitis), we did not expect an association with any extrapulmonary involvement [25]. It is unclear what the underlying pathways and mechanisms could be. We found only one study (on the ACCESS dataset) reporting a similar association, finding that occupational insecticide exposure combined with HLA class II allele DRB1*1101 was associated with cardiac sarcoidosis [3].

### Infectious agents

Our analysis showed that contact with (mammalian) livestock (including cows, goats, sheep, horses) and jobs with close human contact (including health care workers, educators, child and elderly care workers) were independently associated with liver and spleen involvement (Table 3). Moreover, contact with livestock was related to cardiac involvement (OR 9.86; 95% CI 1.95–49.0). We speculate that such contacts entail an increased risk of exposure to infectious agents.

Infectious agents—such as mycobacteria and *Cutibacterium acnes*—have been suspected of being involved in the development of sarcoidosis in some patients [8]. This does not necessarily imply that sarcoidosis is an infection. Numerous researchers have unsuccessfully attempted to culture mycobacteria from sarcoid tissues [26]. Nevertheless, T-cell responses to mycobacterial antigens, such as mKatG, and heat-killed *C. acnes* have been demonstrated in peripheral blood mononuclear cells (PBMCs) and bronchoalveolar lavage fluid of some patients [27, 28].

Interestingly, Beijer et al*.* demonstrated that sarcoidosis patients whose PBMCs responded to mycobacterial antigens, had more cardiac involvement (3/5 patients) than unresponsive patients (34/196; p = 0.044) [29]. Also, patients where *C. acnes* was present in histological samples—confirmed by immunohistochemistry—were more likely to have liver involvement (19% compared to 4%; p = 0.057*)* [30]. Although a systemically disseminated trigger could be suspected, we can only speculate on the agents to which our patients were exposed and the precise mechanisms leading to liver, spleen and/or heart involvement. Unlike in our study, no increase in extrapulmonary sarcoidosis was found in health care or childcare workers in the ACCESS study [12]. Indirect support for the association between close human contact and extrapulmonary sarcoidosis is suggested by studies showing more extrapulmonary sarcoidosis in women, who are more likely to have care jobs than men [10, 11].

We also found an association between administrative jobs and splenic involvement. Although associations between mould exposure in damp indoor environments and sarcoidosis have been reported [9], we could not assess if mould exposure was present in our patients with administrative jobs. In one outbreak of sarcoidosis in office workers in a water-damaged building, 3 out of 6 cases had “multiorgan” sarcoidosis (without further details reported) [31]. In contrast, in the ACCESS dataset, exposure to moulds or musty odours combined with HLA class II allele DRB1*1101 was associated with pulmonary-only sarcoidosis.

### Smoking

In our study, smoking was associated with ocular sarcoidosis (OR 3.26; 95% CI 1.33–7.79) and skin granulomas—although not statistically significant (OR 2.50; 95% CI 0.89–6.54). While smokers are less likely to be diagnosed with sarcoidosis in general [32], smoking has been previously shown to be a risk factor for *ocular* sarcoidosis [33]. It is unclear whether this results from a local effect—with the eye as portal of entry of the disease trigger—or whether it represents a systemic effect.

We could not identify other studies investigating the relation between smoking and skin granulomas in sarcoidosis. Although it is possible that this relation is confounded by other unknown exposures, studies have shown that smoking leads to an increased prevalence of various cutaneous disorders characterized by defective permeability, such as eczema and psoriasis [34]. Smoking might therefore facilitate the penetration of unknown antigens triggering skin granulomas. No associations between the other studied exposures and skin granulomas were found, possibly because we did not specifically assess *dermal* exposures.

### Strengths and limitations

A concern might be that some included patients represent misdiagnosed cases of hypersensitivity pneumonitis (in those exposed to organic dust or reactive chemicals), pneumoconiosis (in those exposed to inorganic dust), or infections. Nevertheless, all included patients had histologically confirmed sarcoid granulomas (with negative cultures and stains, and exclusion of silicotic nodules) or presented with a Löfgren syndrome—highly supportive for a diagnosis of sarcoidosis [15].

One could argue that because sarcoidosis is “by definition” a disease of unknown cause, finding a potential cause excludes the diagnosis of sarcoidosis and requires another disease label. For example, for sarcoidosis cases who had been exposed to WTC dust, Izbicki et al*.* proposed the term “sarcoid-like granulomatous pulmonary disease” since they rarely had extrapulmonary involvement [6]. However, as already argued by Scadding in 1960 [35], this approach is not helpful when investigating the etiology of sarcoidosis, because it would be unclear when the presence of an exposure—epidemiologically related to sarcoidosis—should lead to exclusion from the category "sarcoidosis" and when it should be ignored as incidental and unrelated.

Our study has several limitations. Since the exposure information available from the medical records was not standardized, exposure misclassification is possible. Nevertheless, thanks to the longstanding presence of an outpatient clinic for environmental and occupational disorders within the hospital’s Department of Respiratory Diseases, there is a tradition of routinely registering occupational and environmental histories in the medical records of new sarcoidosis patients [36]. The exposure assessment categories were rather broad because specific agents would have been difficult for experts to assess and would have limited the power of the study. Therefore, our approach possibly obscured the effect of specific exposures, such as certain metals—included in the category “inorganic dust”. Nevertheless, we consider the blinded exposure assessment as a strength of the study.

Because the exposure assessment was done retrospectively, we were unable to reliably estimate the latency period between exposure and disease onset or the precise duration of the exposures. The time relationship between exposure and occurrence of sarcoidosis has barely been studied. Case studies reporting on patients exposed to silica/silicates suggest latency periods from 6 months up to 40 years between exposure and onset of symptoms [37–39]. In a cohort of WTC first responders, a peak incidence of sarcoidosis was found 7–9 years after the WTC collapse [40]. Also, studies looking at duration of exposure and occurrence of sarcoidosis are scarce, and do not show a clear minimally needed duration of exposure [41].

Our recruitment strategy led to selection of patients with pulmonary involvement. In the workup of patients visiting our clinic, screening for extrapulmonary involvement is routinely performed [15]. Although we cannot exclude that subclinical organ involvements were missed, we are confident that we detected clinically relevant involvements, as the distribution of organ involvements in our study was similar to the one found in the ACCESS study [42]. However, limited inclusion of some rare organ manifestations, such as neurological involvement, prevented inclusion in our statistical analysis. Also, because we do not have follow-up data, we were unable to describe the disease course of our patient.

Since smoking and exposure to reactive chemicals, inorganic or organic dust might lead to respiratory health effects independently of the presence of sarcoidosis, exposed patients conceivably seek medical care earlier than unexposed. To assess whether respiratory symptoms potentially confound the association between exposure and organ involvement, we performed a sensitivity analysis by adjusting for lung function parameters. This did not substantially alter the effect size estimates for the different exposures, suggesting the absence of substantial confounding (see Additional file 1: Table S3).

## Conclusion

Our study indicates that, in susceptible individuals, different exposures might be related to different clinical presentations of sarcoidosis. As this association has hardly been investigated, confirmation in other populations is warranted, preferably including more patients with rare organ manifestations. Future longitudinal studies could clarify whether not only disease presentation but also prognosis is related to exposure and if stopping or reducing these exposures could alter the disease course [38].

## Supplementary Information

**Additional file 1: Table S1.** Comorbidities and other covariates of the included sarcoidosis patients; **Table S2.** Results for the univariable analysis of the associations between each organ involvement and each exposure. **Table S3.** Sensitivity analysis to assess potential confounding by adjusting for different lung function parameters in the best-fit logistic regression models from Table 1.

## Data Availability

The datasets during and/or analysed during the current study available from the corresponding author on reasonable request.

## Abbreviations

ACCESS: A Case Control Etiologic Study of Sarcoidosis
AIC: Akaike information criterion
CI: Confidence interval
FEV_1_: Forced expiratory volume in 1 s
FVC: Forced vital capacity
HLA: Human leukocyte antigen
OR: Odds ratio
PBMCs: Peripheral blood mononuclear cells
%pred.: Percentage of the predicted value
TLC: Total lung capacity
T_LCO_: Transfer factor of the lung for carbon monoxide
TNF: Tumor necrosis factor
WASOG: World Association of Sarcoidosis and other Granulomatous Disorders
WTC: World Trade Centre
