# Supplementary material for: Associations between occupational and environmental exposures and organ involvement in sarcoidosis: a retrospective case-case analysis
Source: Respir Res. 2021 Aug 9;22:224. doi: 10.1186/s12931-021-01818-5 (PMC8351152; doi:10.1186/s12931-021-01818-5)
Supplement: Supplementary file 1 — Additional file 1: Table S1. Comorbidities and other covariates of the included sarcoidosis patients; Table S2. Results for the univariable analysis of the associations between each organ involvement and each exposure. Table S3. Sensitivity analysis to assess potential confounding by adjusting for different lung function parameters in the best-fit logistic regression models from Table 1. [file 12931_2021_1818_MOESM1_ESM.docx]

**Additional file 1**

**Title: Associations between occupational and environmental exposures and organ involvement in sarcoidosis: a retrospective case-case analysis**

**Authors:** Steven Ronsmans^1,2^, MD; Jolien De Ridder^3^, MD; Eline Vandebroek^1,4^, MD; Stephan Keirsbilck^1,5^, MD; Benoit Nemery^1,2^, MD, PhD; Peter H M Hoet^2^, PhD; Steven Vanderschueren^6,7^, MD, PhD; Wim A Wuyts^3,8^, MD, PhD; Jonas Yserbyt^3,8^, MD, PhD

*Table S1. Comorbidities and other covariates of the included sarcoidosis patients*

|  |  |  |  |  |  | **Exposure** |  |  |  |
| --- | --- | --- | --- | --- | --- | --- | --- | --- | --- |
|  |  | **Overall** (n = 238) | **Reactive chemicals** (n = 26) | **Inorganic dust** (n = 74) | **Organic dust** (n = 63) | **Contact with livestock** (n = 15) | **Close human contact** (n = 31) | **Admin work only** (n = 42) | **Active smoker**  (n = 43) |
| Systemic autoimmune disease | | 4 (1.7%) | 1 (3.8%) | 1 (1.4%) | 1 (1.6%) | 1 (6.7%) | 0 (0%) | 1 (2.4%) | 1 (2.3%) |
| Organ-specific autoimmune disease | | 14 (5.9%) | 1 (3.8%) | 2 (2.7%) | 4 (6.3%) | 1 (6.7%) | 1 (3.2%) | 2 (4.8%) | 4 (9.3%) |
| Taking medication (before diagnosis) that could have potentially triggered sarcoidosis | | 4 (1.7%) | 0 (0%) | 1 (1.4%) | 0 (0%) | 0 (0%) | 0 (0%) | 2 (4.8%) | 1 (2.3%) |
| Silicone breast implant | | 3 (1.3%) | 0 (0%) | 0 (0%) | 1 (1.6%) | 0 (0%) | 0 (0%) | 1 (2.4%) | 0 (0%) |
| Metal joint implant | | 2 (0.8%) | 0 (0%) | 1 (1.4%) | 2 (3.2%)^§^ | 1 (6.7%) | 0 (0%) | 0 (0%) | 0 (0%) |
| Familial history of sarcoidosis | | 14 (5.9%) | 2 (7.7%) | 5 (6.8%) | 8 (13%)* | 2 (13%) | 0 (0%) | 2 (4.8%) | 1 (2.3%) |
| Familial history of autoimmune of autoinflammatory disease | | 18 (7.6%) | 2 (7.7%) | 1 (1.4%)* | 5 (7.9%) | 0 (0%) | 3 (9.7%) | 7 (17%)* | 5 (12%) |
| Genetic disorder | | 4 (1.7%) | 1 (3.8%) | 1 (1.4%) | 1 (1.6%) | 1 (6.7%) | 2 (6.5%)^§^ | 1 (2.4%) | 1 (2.3%) |
| Statistics presented: n (%); Statistical tests performed, comparing exposed to non-exposed: Fisher's exact test or Pearson's Chi-squared test; * when p<0.05, ^§^ p<0.10 | | | | | | | | | |

*Table S2: Results for the univariable analysis of the associations between each organ involvement and each exposure, without adjustment for other exposures or covariates.*

| **Exposure** | **Pulmonary only**  **(n = 164)** | | | **Liver involvement**  **(n = 23)** | | | **Splenic involvement**  **(n = 37)** | | | **Cardiac involvement**  **(n = 17)** | | | **Eye involvement**  **(n = 29)** | | | **Skin granulomas**  **(n = 23)** | | |
| --- | --- | --- | --- | --- | --- | --- | --- | --- | --- | --- | --- | --- | --- | --- | --- | --- | --- | --- |
|  | **OR*** | **95% CI*** | **p*** | **OR*** | **95% CI*** | **p*** | **OR*** | **95% CI*** | **p*** | **OR*** | **95% CI*** | **p*** | **OR*** | **95% CI*** | **p*** | **OR*** | **95% CI*** | **p*** |
| Reactive chemicals (n = 26) | 0.84 | 0.36 - 2.05 | 0.68 | 0.35 | 0.02 - 1.76 | 0.31 | 0.68 | 0.16 - 2.11 | 0.55 | 3.97 | 1.17 - 11.9 | **0.017** | 0.57 | 0.09 - 2.08 | 0.46 | 0.76 | 0.12 - 2.81 | 0.72 |
| Inorganic dust (n = 74) | 1.98 | 1.06 - 3.85 | **0.036** | 0.30 | 0.07 - 0.92 | 0.061 | 0.38 | 0.14 - 0.89 | **0.039** | 0.45 | 0.10 - 1.44 | 0.22 | 0.67 | 0.26 - 1.59 | 0.39 | 0.76 | 0.27 - 1.93 | 0.59 |
| Organic dust (n = 63) | 1.46 | 0.77 - 2.85 | 0.26 | 1.55 | 0.60 - 3.78 | 0.34 | 0.60 | 0.23 - 1.38 | 0.26 | 0.35 | 0.05 - 1.29 | 0.17 | 0.41 | 0.12 - 1.10 | 0.11 | 1.55 | 0.60 - 3.78 | 0.34 |
| Contact with livestock (n = 15) | 0.27 | 0.09 - 0.79 | **0.018** | 3.90 | 1.01 - 12.7 | **0.031** | 4.13 | 1.31 - 12.3 | **0.012** | 5.87 | 1.47 - 20.0 | **0.006** | 0.00 |  | 0.99 | 0.65 | 0.04 - 3.49 | 0.69 |
| Close human contact (n= 31) | 0.43 | 0.20 - 0.92 | **0.029** | 4.45 | 1.64 - 11.5 | **0.002** | 3.17 | 1.31 - 7.36 | **0.008** | 0.88 | 0.13 - 3.35 | 0.87 | 1.92 | 0.66 - 4.93 | 0.20 | 0.61 | 0.09 - 2.24 | 0.52 |
| Administrative work only (n = 42) | 0.78 | 0.39 - 1.59 | 0.48 | 0.98 | 0.27 - 2.79 | 0.97 | 1.64 | 0.68 - 3.68 | 0.25 | 1.48 | 0.40 - 4.45 | 0.51 | 0.97 | 0.31 - 2.52 | 0.95 | 0.68 | 0.15 - 2.10 | 0.54 |
| Active smoker (n = 43) | 1.39 | 0.67 - 3.05 | 0.39 | 0.66 | 0.15 - 2.03 | 0.51 | 0.50 | 0.14 - 1.36 | 0.22 | 0.97 | 0.22 - 3.14 | 0.96 | 3.38 | 1.43 - 7.76 | **0.004** | 2.18 | 0.79 - 5.50 | 0.11 |
| ** OR = Odds Ratio for having been exposed (cases with a given organ involvement versus all other cases). CI = Confidence Interval;* **p-value*; bold when p<0.05*** | | | | | | | | | | | | | | | | | | |

*Table S3. Sensitivity analysis to assess potential confounding by adjusting for different lung function parameters in the best-fit logistic regression models from Table 1.*

| **Exposure** | **Pulmonary only**  **(n = 164)** | | | **Liver involvement**  **(n = 23)** | | | **Splenic involvement**  **(n = 37)** | | | **Cardiac involvement**  **(n = 17)** | | | **Eye involvement**  **(n = 29)** | | | **Skin granulomas**  **(n = 23)** | | |
| --- | --- | --- | --- | --- | --- | --- | --- | --- | --- | --- | --- | --- | --- | --- | --- | --- | --- | --- |
|  | **OR*** | **95% CI*** | **p*** | **OR*** | **95% CI*** | **p*** | **OR*** | **95% CI*** | **p*** | **OR*** | **95% CI*** | **p*** | **OR*** | **95% CI*** | **p*** | **OR*** | **95% CI*** | **p*** |
| Reactive chemicals (n = 26) |  |  |  |  |  |  |  |  |  | 4.13 | 0.96 - 16.5 | **0.047** |  |  |  |  |  |  |
| Inorganic dust (n = 74) | 2.22 | 1.14 - 4.50 | **0.022** |  |  |  |  |  |  | 0.24 | 0.05 - 0.88 | **0.045** |  |  |  |  |  |  |
| Organic dust (n = 63) | 1.95 | 0.96 - 4.17 | 0.074 |  |  |  |  |  |  | 0.15 | 0.02 - 0.69 | **0.031** |  |  |  |  |  |  |
| Contact with livestock (n = 15) | 0.15 | 0.04 - 0.52 | **0**.**004** | 4.08 | 0.97 - 14.7 | **0.038** | 7.57 | 2.05 - 28.4 | **0.002** | 11.6 | 2.11 - 63.7 | **0.004** | 0.00 |  | >0.99 |  |  |  |
| Close human contact (n= 31) |  |  |  | 4.21 | 1.51 - 11.2 | **0.004** | 4.23 | 1.58 - 11.1 | **0.003** |  |  |  |  |  |  |  |  |  |
| Administrative work only (n = 42) |  |  |  |  |  |  | 3.04 | 1.14 - 7.91 | **0.023** |  |  |  |  |  |  |  |  |  |
| Active smoker (n = 43) |  |  |  |  |  |  |  |  |  |  |  |  | 2.39 | 0.90 - 6.08 | 0.071 | 2.05 | 0.66 - 5.76 | 0.2 |
| FEV_1_ %pred | 1.01 | 0.99 - 1.03 | 0.4 | 1.00 | 0.97 - 1.03 | >0.9 | 0.99 | 0.97 - 1.02 | 0.6 | 0.99 | 0.95 - 1.03 | 0.6 | 1.02 | 0.99 - 1.05 | 0.14 | 1.01 | 0.98 - 1.04 | 0.6 |
| FEV_1_/FVC % | 1.00 | 0.96 - 1.04 | 0.8 | 1.02 | 0.97 - 1.09 | 0.4 | 1.01 | 0.96 - 1.06 | 0.8 | 0.98 | 0.91 - 1.05 | 0.5 | 0.97 | 0.92 - 1.03 | 0.3 | 0.98 | 0.92 - 1.05 | 0.6 |
| *T*_LCO_ %pred | 1.00 | 0.98 - 1.02 | 0.9 | 0.99 | 0.96 - 1.03 | 0.7 | 0.99 | 0.96 - 1.01 | 0.3 | 1.01 | 0.98 - 1.05 | 0.4 | 0.98 | 0.95 - 1.00 | 0.10 | 1.00 | 0.97 - 1.03 | >0.9 |
| ** OR = Odds Ratio for having been exposed (cases with a given organ involvement versus all other cases) while adjusting for other exposures and covariates (see Study design and methods); For lung function parameters the OR is given per percent of the predicted value; CI = Confidence Interval;* ***p-value; bold when statistically significant (*p*<0.05)*** | | | | | | | | | | | | | | | | | | |
